# Disseminated Nocardia farcinica Infection Presenting as Multiple Intramuscular Abscesses Mimicking Skeletal Muscle Metastases in a Patient With Hepatocellular Carcinoma

**DOI:** 10.7759/cureus.114166

**Published:** 2026-08-08

**Authors:** Akitoshi Kanagawa, Tomohiro Itonaga, Tatsuhiko Zama, Ryuji Mikami, Kazuhiro Saito

**Affiliations:** 1 Department of Radiology, Tokyo Medical University, Tokyo, JPN

**Keywords:** brain abscess, compartment syndrome, hepatocellular carcinoma, immunocompromised host, nocardia farcinica, nocardiosis, radiotherapy complication, skeletal muscle metastasis

## Abstract

A 67-year-old man with hepatocellular carcinoma and a previously resected pulmonary metastasis was receiving prednisolone for immune-related interstitial nephritis. Two months after pulmonary metastasectomy, multiple pulmonary nodules and painful intramuscular masses developed in the right forearm, left gluteus medius, and left obturator internus. The lesions were initially interpreted as metastases, and palliative radiotherapy was initiated. At the initial radiation oncology consultation, the patient was afebrile but had right forearm edema, neutrophilic leukocytosis, and an elevated C-reactive protein level. After 21 Gy in seven fractions to the right forearm, rapidly progressive swelling and pain led to emergency decompression for acute compartment syndrome. A purulent collection was identified; blood culture grew *Nocardia farcinica*, and direct smear examination of aspirated fluid showed branching filamentous Gram-positive bacteria consistent with *Nocardia* species. Subsequent brain magnetic resonance imaging demonstrated multiple cerebral abscesses. Radiotherapy was discontinued, and the patient improved after drainage and prolonged combination antimicrobial therapy. This case demonstrates that multifocal nocardial infection may be mistaken for metastatic disease in a corticosteroid-treated patient with cancer and emphasizes the need to integrate physical examination, laboratory abnormalities, and imaging findings before treating unbiopsied soft-tissue lesions.

## Introduction

*Nocardia* species are aerobic, partially acid-fast, branching, Gram-positive bacilli widely distributed in soil, dust, and decaying organic matter [1]. Infection usually occurs through inhalation or direct cutaneous inoculation and may disseminate hematogenously to the central nervous system, skin, and soft tissues [1]. Nocardiosis primarily affects patients with impaired cell-mediated immunity, including those receiving corticosteroids or other immunosuppressive therapies [2]. Because *Nocardia* is acquired directly from environmental exposure rather than through person-to-person transmission, no vaccine or established antimicrobial prophylaxis exists for at-risk patients; the principal safeguard is heightened clinical vigilance for atypical soft-tissue or pulmonary lesions during corticosteroid or other immunosuppressive therapy. Its clinical and imaging manifestations are nonspecific and may mimic malignancy, tuberculosis, or other opportunistic infections [1,2]. In patients with a known malignancy, multiple enlarging lesions may be attributed to metastatic disease, and this diagnostic anchoring can delay the recognition of infection [3,4]. Among *Nocardia* species, *N. farcinica* is of particular clinical importance: it is more often resistant to first-line antimicrobial agents and has a stronger predilection for hematogenous spread to the central nervous system than most other *Nocardia* species, which underscores the importance of species-level identification [5]. We report a patient with hepatocellular carcinoma (HCC) in whom disseminated *N. farcinica* caused multiple intramuscular abscesses that were initially interpreted as skeletal muscle metastases - a presentation that is inherently atypical, as skeletal muscle metastasis occurs in only an estimated 0%-1.5% of patients with HCC [6] - leading to palliative radiotherapy before the infectious diagnosis was established. We describe the clinical course and imaging findings and discuss features that may help distinguish nocardial abscesses from metastatic lesions.

## Case presentation

History and initial presentation

**Figure 1:**
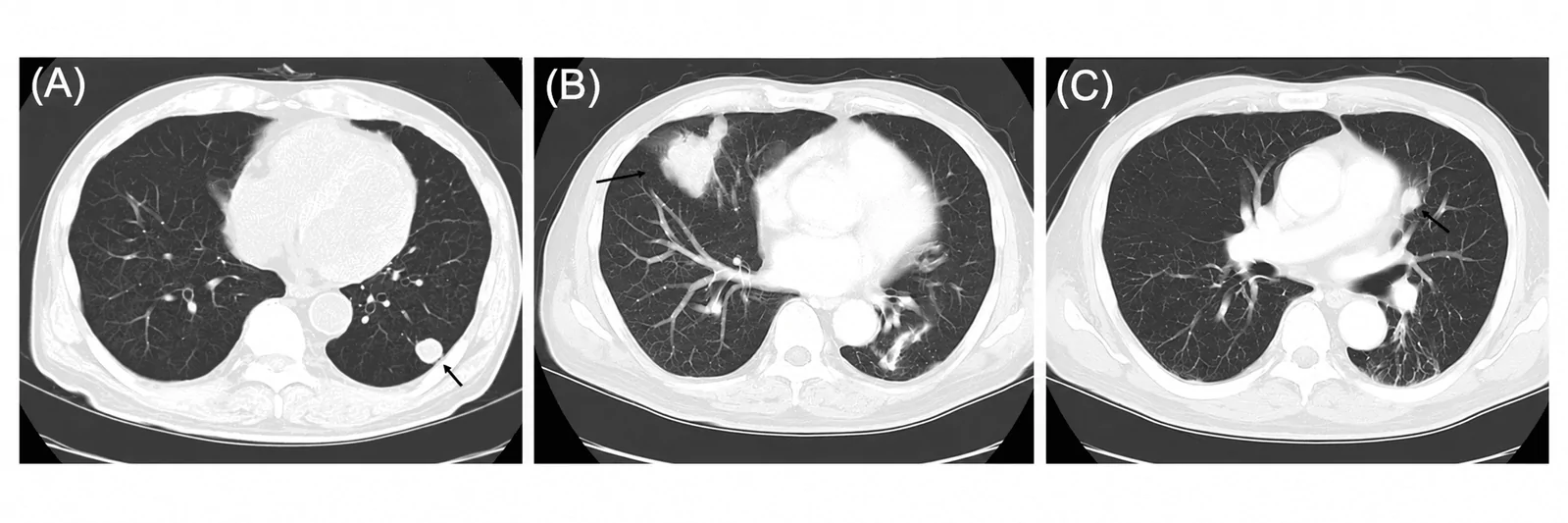
Chest CT Scans Two Months Prior to and at the Time of Radiation Oncology Consultation Chest CT images obtained two months before and at the radiation oncology consultation. (A) CT image obtained two months before consultation shows a left lower-lobe nodule (arrow), which was surgically resected and confirmed as a metastasis from hepatocellular carcinoma (HCC). (B, C) CT images at consultation show new nodules in the right middle lobe (B) and the lingula of the left lung (C) (arrows).

**Figure 2:**
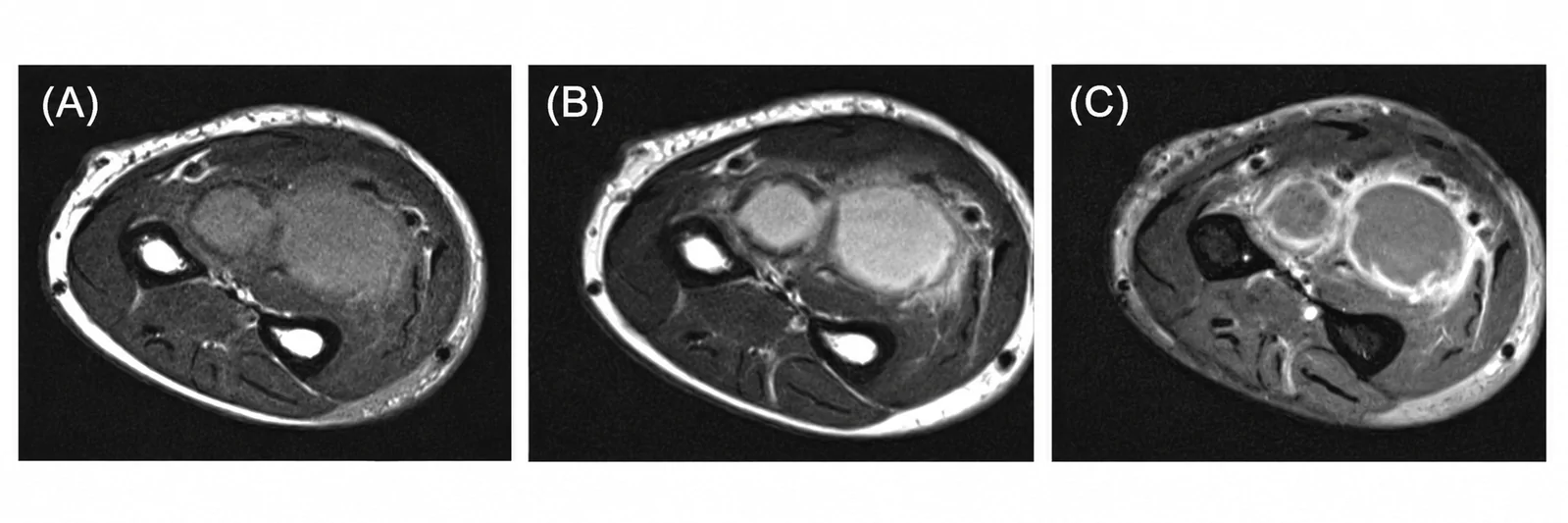
MRI Findings of the Right Forearm Mass Right forearm MRI at presentation. (A) Axial T1-weighted image shows a mildly hyperintense intramuscular lesion (arrows). (B) Axial T2-weighted image shows marked lesion hyperintensity (arrows) with extensive surrounding edema. (C) Axial fat-suppressed contrast-enhanced image shows peripheral rim enhancement of the lesion (arrows) and enhancement in the surrounding edematous tissue.

A 67-year-old man presented with progressive right forearm pain of two months' duration. Five years earlier, he had undergone partial hepatectomy for solitary HCC. One year after surgery, a solitary pulmonary metastasis was detected in the left lung, and systemic therapy with atezolizumab and bevacizumab was initiated. Four months before the current presentation, systemic therapy was discontinued because of immune-related interstitial nephritis, and prednisolone was started at 50 mg/day and tapered to 20 mg/day. Because the pulmonary metastasis persisted, partial resection of the left lower lobe was performed two months before presentation. Histopathologic examination confirmed metastatic HCC with a close resection margin. The alpha-fetoprotein L3 fraction (AFP-L3) decreased after resection; protein induced by vitamin K absence or antagonist-II (PIVKA-II) initially decreased but subsequently increased. Follow-up computed tomography (CT) demonstrated multiple new pulmonary nodules (Figure 1) and intramuscular masses (Figure 2). He was referred to the Department of Radiation Oncology for palliation of right forearm pain. Physical examination revealed right forearm edema and tenderness and left gluteal pain that worsened while sitting; no erythema, warmth, or fluctuance was present. His body temperature was 36.9°C. Laboratory testing showed an elevated white blood cell (WBC) count with neutrophilia and lymphopenia, an elevated C-reactive protein (CRP) level, hypoalbuminemia, and an elevated PIVKA-II level; renal function was preserved (Table 1).

**Table 1:** Laboratory findings at the initial radiation oncology consultation and at the onset of compartment syndrome. *For eGFR, ≥60 mL/min/1.73 m² is a clinical decision threshold rather than a formal biological reference interval. Reference ranges are those used by the Central Clinical Laboratory, Tokyo Medical University Hospital. WBC: white blood cell; CRP: C-reactive protein; eGFR, estimated glomerular filtration rate; LDH, lactate dehydrogenase; CK, creatine kinase; AFP-L3: isoform of alpha-fetoprotein; PIVKA-II; protein induced by vitamin K absence or antagonist-II; -, data not available.

Laboratory parameter	Reference range	Initial consultation	Onset of compartment syndrome
WBC count (×10³/µL)	3.3-8.6	14	21
Neutrophils (%)	42.0-74.0	88.5	89.2
Lymphocytes (%)	19.0-47.0	5.1	4.4
CRP (mg/dL)	0.00-0.14	6.03	16.3
Serum creatinine (mg/dL)	0.65-1.07 (men)	0.79	0.84
eGFR (mL/min/1.73 m²)	≥60*	75.1	70.2
Albumin (g/dL)	4.1-5.1	2.7	2.5
LDH (U/L)	124-222	212	189
CK (U/L)	59-248 (men)	34	108
AFP-L3 (%)	<10.0	<0.5	-
PIVKA-II (mAU/mL)	<40	230	-

Imaging findings at presentation

CT demonstrated new nodules in the right middle lobe and the lingula of the left lung (Figure 1). New intramuscular masses were present in the right forearm, left obturator internus, and left gluteus medius (Figure 2). Magnetic resonance imaging (MRI) of the right forearm showed faint T1 hyperintensity and marked T2 hyperintensity, with surrounding edema on T2-weighted images. Fat-suppressed contrast-enhanced MRI demonstrated peripheral rim enhancement (Figure 2). Given the recent pathologically confirmed pulmonary metastasis, the simultaneous appearance of multiple pulmonary and intramuscular lesions, and the renewed elevation of PIVKA-II, the lesions were collectively interpreted as metastatic disease.

Course of radiotherapy and complications

**Figure 3:**
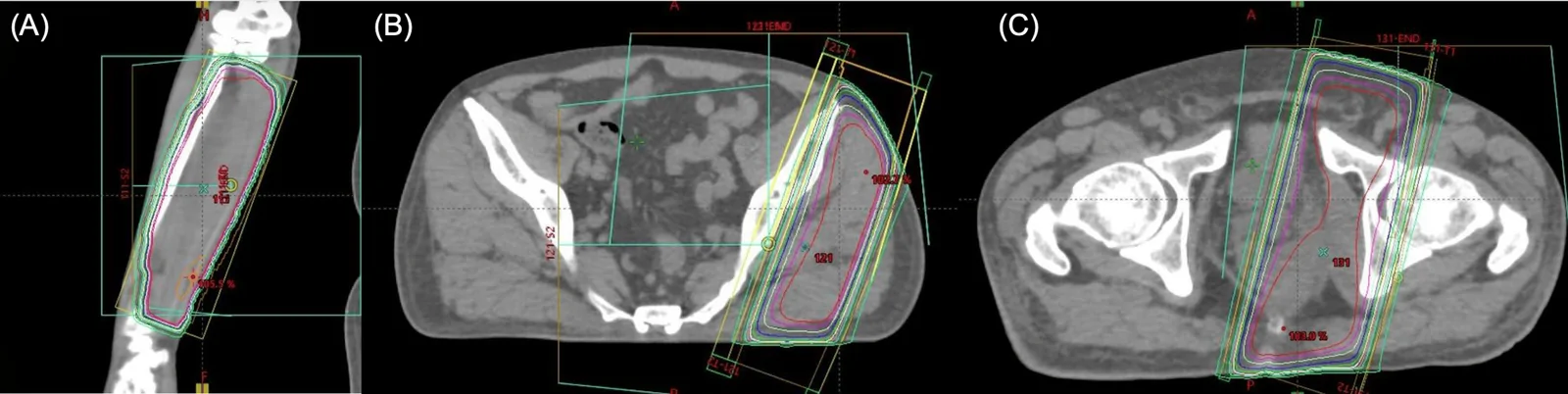
Radiotherapy Treatment Planning CT for the Three Presumed Metastatic Sites Radiotherapy planning CT with dose distributions for the three lesions treated as presumed skeletal muscle metastases: (A) right forearm, (B) left gluteus medius, and (C) left obturator internus. A total dose of 30 Gy in 10 fractions was planned for each site. Treatment to the right forearm was discontinued after 21 Gy in seven fractions when compartment syndrome developed; treatment to the left gluteus medius and left obturator internus was discontinued after 27 Gy in nine fractions when nocardiosis was suspected.

**Figure 4:**
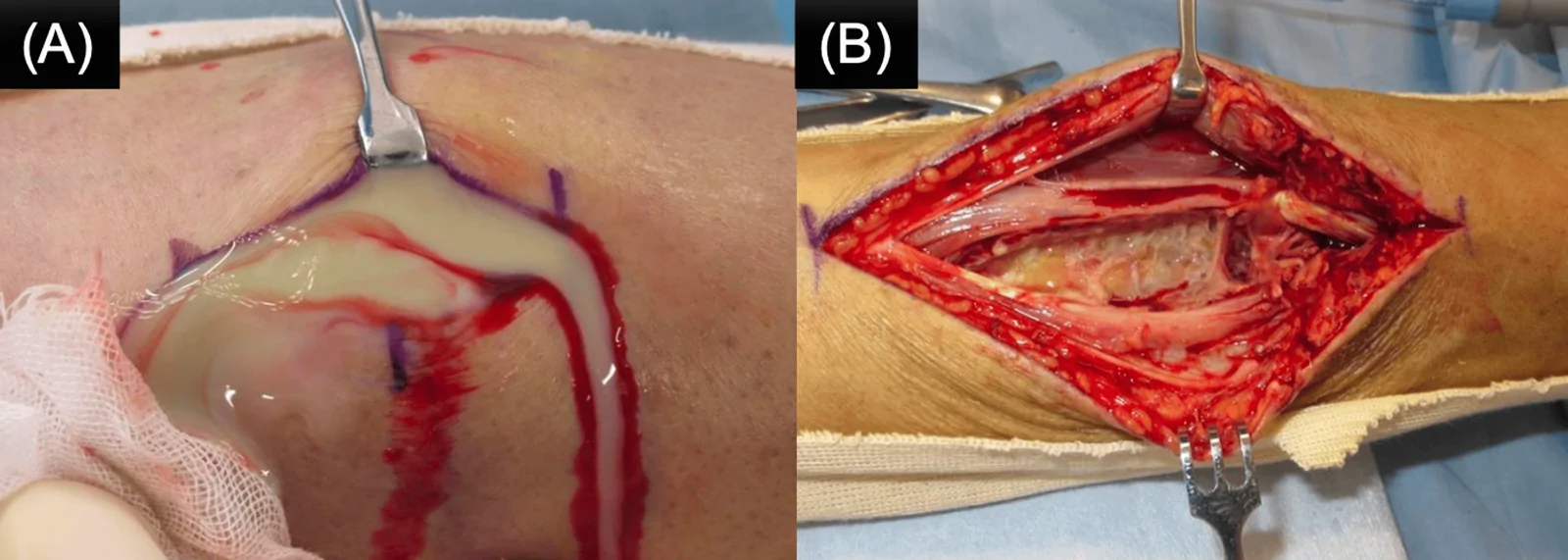
Intraoperative Findings During Emergency Decompression and Drainage of the Right Forearm Intraoperative photographs obtained during emergency decompression and drainage of the right forearm for acute compartment syndrome. (A, B) A cream-colored purulent collection is present within the involved compartment. Histopathologic examination showed no malignant cells, and direct smear examination of aspirated fluid demonstrated branching filamentous Gram-positive organisms consistent with *Nocardia* species.

Palliative radiotherapy of 30 Gy in 10 fractions (3 Gy per fraction) was planned for the right forearm, left gluteus medius, and left obturator internus lesions (Figure 3). After 21 Gy in seven fractions to the right forearm, swelling and pain rapidly worsened, and acute compartment syndrome developed. His body temperature was 37.1°C. The WBC count and CRP level had increased further, whereas creatine kinase and serum creatinine remained within their reference ranges (Table 1). The preserved creatine kinase level argued against significant myonecrosis, suggesting that the rise in compartment pressure reflected abscess expansion and surrounding inflammatory edema rather than a primary muscle-injury process. Two sets of blood cultures were obtained, and emergency surgical decompression and drainage were performed by the Department of Orthopedic Surgery. A cream-colored purulent collection rather than a solid tumor was found intraoperatively (Figure 4), and histopathologic examination showed no malignant cells. Radiotherapy to the right forearm was discontinued. While microbiologic results were pending, radiotherapy to the left gluteus medius and left obturator internus continued; both sites received 27 Gy in nine fractions before treatment was stopped. After four days, one of four blood-culture bottles became positive and ultimately grew *N. farcinica*. Direct smear examination of aspirated abscess fluid demonstrated branching filamentous Gram-positive bacteria consistent with *Nocardia* species.

Treatment and outcome

**Figure 5:**
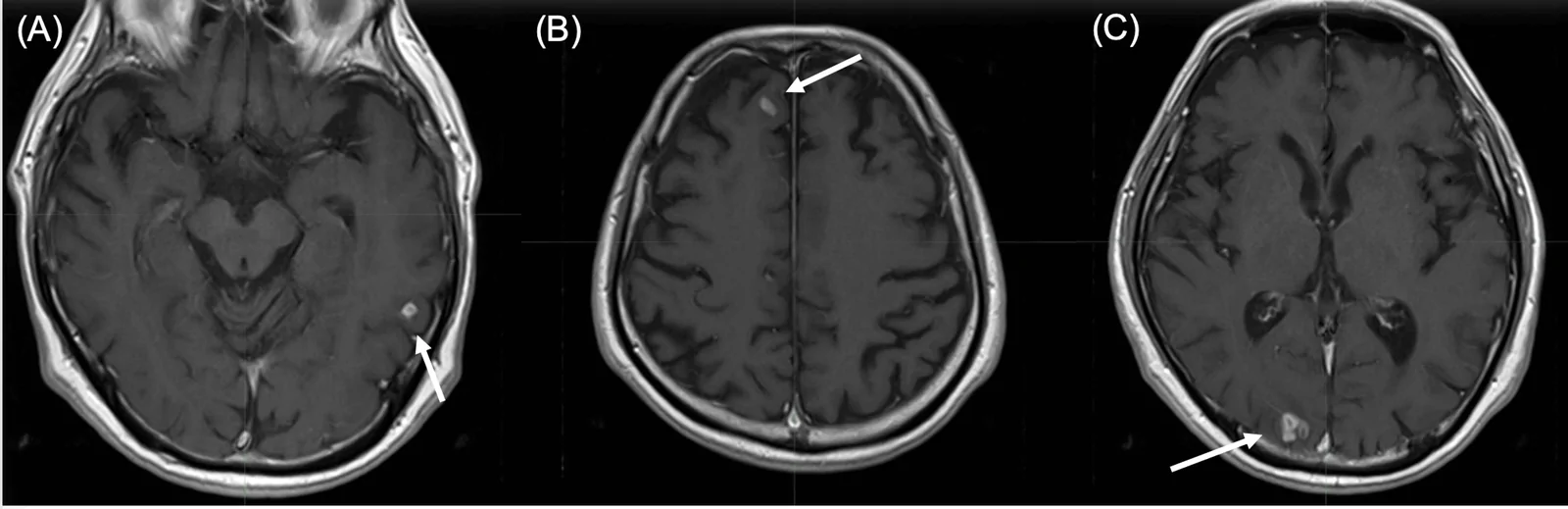
Contrast-Enhanced Brain MRI Showing Multiple Cerebral Abscesses Contrast-enhanced axial T1-weighted brain MRI obtained 27 days after starting radiotherapy. (A-C) Multiple variably sized ring-enhancing nodules are present in both cerebral hemispheres, consistent with cerebral abscesses (arrows).

Disseminated nocardiosis was diagnosed based on the isolation of *N. farcinica* from blood culture, identified by matrix-assisted laser desorption/ionization time-of-flight mass spectrometry (MALDI-TOF MS), and *Nocardia*-compatible organisms in the aspirated fluid. Bacterial culture of the aspirated forearm fluid showed no growth, and the diagnosis at that site therefore rested on smear morphology rather than culture confirmation. The left gluteus medius/obturator internus lesion was not directly sampled. Because the forearm culture did not yield growth, antimicrobial susceptibility testing could not be performed on the abscess isolate. Empiric combination therapy was therefore selected on the basis of blood-culture species identification and published treatment guidance rather than site-specific susceptibility data. Because *N. farcinica* frequently involves the central nervous system [5], contrast-enhanced brain MRI was performed 27 days after radiotherapy began. T1-weighted images showed multiple variably sized ring-enhancing nodules consistent with cerebral abscesses (Figure 5). Treatment comprised trimethoprim-sulfamethoxazole, imipenem-cilastatin, and amikacin, together with surgical drainage of the right forearm abscess. The patient improved and was discharged 45 days after antimicrobial therapy began. On interval follow-up, the right forearm abscess cavity, assessed by CT, had become nearly indiscernible by antimicrobial treatment day 56. Follow-up CT on day 141 showed that all pulmonary nodules had become indistinct and that the left gluteus medius and obturator internus lesions had resolved. The cerebral abscesses, assessed by contrast-enhanced MRI, were nearly completely resolved by day 154, indicating marked radiologic improvement at all the involved sites. During antimicrobial follow-up, AFP-L3 remained <0.5%, whereas PIVKA-II declined from 230 mAU/mL at the initial consultation to values in the low 20s mAU/mL and remained stable through treatment day 154, supporting an infectious rather than recurrent malignant process. The pulmonary, gluteal/obturator, and cerebral lesions were not directly sampled and were considered presumptive nocardial involvement based on their parallel radiologic resolution during antimicrobial therapy.

Clinical timeline

For clarity, the sequence of events is summarized as follows: referral to the Department of Radiation Oncology for right forearm pain → palliative radiotherapy planned to the right forearm, left gluteus medius, and left obturator internus → after 21 Gy in seven fractions to the right forearm, acute compartment syndrome developed, prompting emergency surgical decompression and drainage → radiotherapy to the right forearm discontinued, while radiotherapy to the left gluteus medius and left obturator internus continued to a total of 27 Gy in nine fractions → four days after cultures were obtained, one of four blood-culture bottles became positive for *N. farcinica*, and radiotherapy to the remaining sites was discontinued → 27 days after radiotherapy began, contrast-enhanced brain MRI revealed multiple cerebral abscesses → antimicrobial therapy was continued, and the patient was discharged 45 days after treatment began → follow-up imaging confirmed progressive resolution of the right forearm abscess (treatment day 56), pulmonary nodules and left gluteus medius and obturator internus lesions (day 141), and cerebral abscesses (day 154).

## Discussion

The diagnostic challenge was that a patient with a recently confirmed pulmonary metastasis from HCC developed multiple pulmonary and soft-tissue lesions that were readily interpreted as metastatic progression. The case is discussed from four perspectives: skeletal muscle metastasis, imaging differentiation, diagnostic anchoring, and implications for radiation oncology practice.

Skeletal muscle metastasis

Skeletal muscle metastases are uncommon in solid tumors; in HCC, the reported incidence is approximately 0%-1.5% [6]. The proposed explanations for the relative resistance of skeletal muscle to metastatic colonization include mechanical disruption of tumor cells by muscle contraction, an unfavorable local pH, high blood flow, and lactate production [6]. Given the recent pathologic confirmation of pulmonary metastasis, the initial attribution of the new lesions to metastatic disease was understandable. However, the rapid transition from a solitary metastatic pattern to multiple painful intramuscular lesions shortly after surgery was atypical and should have prompted diagnostic reassessment.

Diagnostic anchoring and cognitive bias

This case illustrates anchoring bias. The initial impression that new lesions in a patient with HCC represented further malignancy was not adequately revised despite atypical or contradictory findings [3,4]. The rapid appearance of multiple pulmonary nodules after surgery for a lung metastasis, together with the increase in PIVKA-II (protein induced by vitamin K absence or antagonist-II), supported the working diagnosis of widespread metastatic disease. The absence of fever also reduced concern for infection. However, corticosteroid therapy can blunt febrile responses [7], and the combination of forearm edema, progressive pain, neutrophilic leukocytosis, elevated C-reactive protein (CRP), lymphopenia, and rapidly evolving multifocal lesions supported an infectious differential. Avoiding anchoring does not require abandoning the most likely diagnosis; it requires actively seeking discordant findings that could alter management, particularly before treating a lesion without histologic or microbiologic confirmation.

Differential diagnosis of abscess and metastatic tumor

Multiple new pulmonary nodules were initially interpreted as metastases because of the recent pathologically confirmed lung metastasis. In the right forearm, the lesion was mildly hyperintense on T1-weighted images and markedly hyperintense on T2-weighted images, with extensive surrounding T2 hyperintensity, reflecting edema or inflammatory spread. Contrast-enhanced MRI showed a thin, relatively uniform peripheral rim with minimal internal enhancement, a pattern compatible with an abscess but not specific [8]. The pulmonary nodules were also compatible with pulmonary nocardiosis [9]. Dedicated MRI of the lung parenchyma was not performed because chest CT was the clinically preferred modality for evaluating these small pulmonary nodules; consequently, no corresponding pulmonary MR images were available. In retrospect, the integration of these imaging features with the clinical and laboratory findings could have prompted aspiration, biopsy, or microbiologic evaluation before radiotherapy.

Implications for radiation oncology practice

This case offers several lessons for radiation oncologists. First, before radiotherapy for a painful soft-tissue lesion, examination findings including erythema, warmth, fluctuance, edema, skin changes, and tenderness should be documented even when metastasis appears likely. Second, atypical clinical or imaging findings, corticosteroid exposure, or abnormal inflammatory markers should lower the threshold for aspiration, biopsy, and culture. Third, unexpected worsening of pain or swelling during treatment warrants immediate reassessment and consideration of pausing radiotherapy. Although compartment syndrome developed during radiotherapy, temporal association alone does not establish causation; progression of the untreated abscess sufficiently explains the event. Disseminated nocardiosis requires prolonged antimicrobial therapy guided by susceptibility testing when available; initial treatment commonly combines trimethoprim-sulfamethoxazole with a carbapenem and/or amikacin, with drainage of accessible abscesses [10]. In the present case, site-specific susceptibility data were unavailable because the abscess culture showed no growth. Recognition of this mimic is therefore relevant to medical oncologists, radiation oncologists, radiologists, and infectious disease specialists caring for immunosuppressed patients with cancer. The central lesson is that diagnostic anchoring can delay recognition of a treatable infection.

## Conclusions

Disseminated *N. farcinica* infection presented with a microbiologically supported right forearm abscess and presumptive pulmonary, gluteal/obturator, and cerebral involvement, closely mimicking metastatic disease. Blood culture established species-level identification, while direct smear examination of aspirated forearm material supported nocardial involvement at that site despite a negative site-specific culture; the pulmonary, gluteal/obturator, and cerebral lesions were inferred from their parallel radiologic resolution during antimicrobial therapy. Anchoring on metastatic progression led to palliative radiotherapy before the infectious etiology was recognized, and the diagnosis was established after limb-threatening compartment syndrome developed. In patients receiving corticosteroids, the absence of fever does not exclude invasive infection. Rapidly enlarging, painful soft-tissue lesions warrant careful clinical assessment and timely tissue sampling or microbiologic investigation before treatment.
